# *Plasmodium* infection inhibits the expansion and activation of MDSCs and Tregs in the tumor microenvironment in a murine Lewis lung cancer model

**DOI:** 10.1186/s12964-019-0342-6

**Published:** 2019-04-12

**Authors:** Dickson Adah, Yijun Yang, Quan Liu, Kranthi Gadidasu, Zhu Tao, Songlin Yu, Linglin Dai, Xiaofen Li, Siting Zhao, Limei Qin, Li Qin, Xiaoping Chen

**Affiliations:** 1 State Key Laboratory of Respiratory Disease, Center of Infection and Immunity, Guangzhou Institutes of Biomedicine and Health, Chinese Academy of Sciences, 190 Kaiyuan Avenue, Science Park, Guangzhou, 510530 People’s Republic of China; 2 University of Chinese Academy of Sciences, No. 190 Yuquan Road, Beijing, 100049 People’s Republic of China; 3 Guangzhou Regenerative Medicine and Health-Guangdong Laboratory GRMH-GDL, Guangzhou, 510530 People’s Republic of China

**Keywords:** Lung cancer, MDSC, Tregs, Recruiting molecules, PD-1

## Abstract

**Background:**

A major challenge in the development of effective cancer immunotherapy is the ability of tumors and their microenvironment to suppress immune cells through immunosuppressive cells such as myeloid -derived suppressor cells and regulatory T cells. We previously demonstrated that *Plasmodium* infection promotes innate and adaptive immunity against cancer in a murine Lewis lung cancer model but its effects on immunosuppressive cells in the tumor microenvironment are unknown.

**Methods:**

Whole Tumors and tumor-derived sorted cells from tumor-bearing mice treated with or without plasmodium infected red blood cells were harvested 17 days post tumor implantation and analyzed using QPCR, western blotting, flow cytometry, and functional assays. Differences between groups were analyzed for statistical significance using Student’s t-test.

**Results:**

Here we found that *Plasmodium* infection significantly reduced the proportions of MDSCs and Tregs in the lung tumor tissues of the treated mice by downregulating their recruiting molecules and blocking cellular activation pathways. Importantly, CD8^+^ T cells isolated from the tumors of *Plasmodium*-treated mice exhibited significantly higher levels of granzyme B and perforin and remarkably lower levels of PD-1.

**Conclusion:**

We reveal for the first time, the effects of *Plasmodium* infection on the expansion and activation of MDSCs and Tregs with a consequent elevation of CD8^+^T cell-mediated cytotoxicity within the tumor microenvironment and hold great promise for the development of effective immunotherapeutic strategies.

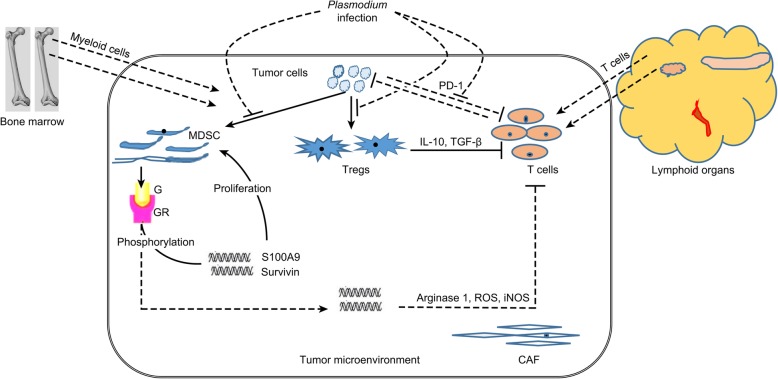

**Electronic supplementary material:**

The online version of this article (10.1186/s12964-019-0342-6) contains supplementary material, which is available to authorized users.

## Background

A key pathological feature of solid cancer is the massive mobilization and infiltration of immune cells into the lymphoid organs and the tumor microenvironment. Solid tumors are known for their ability to recruit and modify immune cells and endothelial cells which form the tumor microenvironment [1]. A growing body of evidence suggests that host immune cells with a suppressive phenotype pose a significant challenge to the success of immune-enhancing anticancer therapy [2–5]. Among these suppressor cells, myeloid-derived suppressor cells (MDSCs) and regulatory T cells (Tregs) have been shown to increase significantly in number in hosts with advanced malignancies [6, 7]. MDSCs are a heterogeneous population of cells that expand during cancer, inflammation and infection, and have a remarkable ability to suppress T-cell responses [8]. There are two main subsets of MDSCs: polymorphonuclear MDSCs (P-MDSCs), which are similar in phenotype and morphology to neutrophils, and monocytic MDSCs (M-MDSCs), which are similar to monocytes [9–11]. In cancer, several differences in distribution and functions have been observed in these MDSC subsets. In the peripheral lymphoid organs, there are more P-MDSCs than M-MDSCs; these P-MDSCs have a relatively modest suppressive activity and play a key role in the regulation of tumor-specific immune responses, facilitating the development of tumor-specific T-cell tolerance. M-MDSCs in the peripheral lymphoid organs lack the capacity to differentiate into macrophages (MØ) and dendritic cells (DC). In contrast, MDSCs in the tumor microenvironment are more suppressive, mostly M-MDSC, and can differentiate into tumor- associated macrophages (TAMs)(reviewed in [12]). This further highlights the importance of novel strategies to inhibit multiple subsets of MDSCs both in the peripheral lymphoid organs and in the tumor microenvironment. Several MDSC-inhibiting drugs have been tested but only against splenic MDSCs, highlighting the need for the development of immune-enhancing drugs and interventions to target MDSCs in the tumor microenvironment.

Growing evidence points to the accumulation of P-MDSC and M-MDSC subsets accompanied by a dynamic change in the macrophages that enter the tumor site toward a predominantly IL10-producing M2 phenotype, all of which have the ability to induce T-regs cells and/or directly suppress antitumor T cell function [13]. In tumor-bearing mice, MDSCs can themselves facilitate the generation of tumor-specific Tregs [14] suggesting that MDSC- targeted inhibition could facilitate the suppression of tumor-specific Treg generation and a resultant increase in the antitumor immune response.

Tregs are a subset of immunosuppressive T cells that are involved in immune homeostasis and self-tolerance, fetal maternal tolerance, infection, autoimmunity, graft-versus-host immunity, and tumor immunity [15]. In the tumor microenvironment, Tregs contribute to the development of immunosuppression, immune evasion and cancer progression [16, 17]. Tregs are recruited to the tumor microenvironment via interactions between tumor-secreted chemokines and their receptors (mainly CCL17/CCL22-CCR4, CCL5-CCR5, CCL28-CCR10 and CXCL9/10/11-CXCR3), converted to a suppressive phenotype by TGF-β, and efficiently expanded in the presence of tumor-derived IL-10 and TGF-β [16]. The accumulation of Tcell-inhibiting [18–20] Tregs in the tumor microenvironment and peripheral blood of cancer patients has been reported in several papers and is correlated with a poor prognosis [21], suggesting that immunotherapeutic approaches targeting Tregs could prolong patient survival.

Although malaria is a serious disease caused by the protozoan genus *Plasmodium*, several reports have suggested that malaria could stimulate the host innate and adaptive immune systems to combat cancer [22–25] by stimulating IFN-γ production, activating natural killer (NK) cells, γ δ T cells, inducing the maturation of DCs and stimulating T-cell proliferation [24, 26].

We have previously demonstrated that *Plasmodium* infection significantly suppresses LLC cell growth via the induction of innate and adaptive antitumor responses in a mouse model [22], but it is not yet known whether *Plasmodium* infection can inhibit the recruitment and activation of MDSCs in the tumor microenvironment. Several studies have been carried out on MDSCs in the peripheral blood of tumor-bearing patients but few studies have focused on tumor-infiltrating MDSCs. The tumor microenvironment is particularly important given that peripheral MDSCs differ from tumor-infiltrating MDSCs in both murine and human cancers [27, 28]. Our current study builds on these findings and further suggests that the induction of innate and adaptive antitumor responses by *Plasmodium* infection was enhanced, at least in part, through the inhibition of MDSCs and Tregs within the tumor microenvironment.

## Materials and methods

### Ethics statement

The animal experiment facilities were approved by the Guangdong Provincial Department of Science and Technology, and complied with the guidelines of the Animal Care Committee, Guangzhou Institutes of Biomedicine and Health, Chinese Academy of Sciences. All efforts were made to minimize animal suffering.

### Sources of animals, cells, and parasites

Six to eight-week old female C57BL/6 mice were purchased from SLAC Laboratory Animal Company (Shanghai, China) and raised in the animal facility of the Guangzhou Institutes of Biomedicine and Health, CAS. The nonlethal *P. yoelli* 17XNL (Py) strain was a donation from the Malaria Research and Reference Reagent Resource Center (MR4). The murine (LLC) cell line was purchased from ATCC and maintained in RPMI 1640 (Gibco, Carlsbad, CA, USA), supplemented with penicillin (80 U/ml), streptomycin (100 U/ml) and 10% FBS in a humidified atmosphere of 5% CO_2_ at 37 °C.

### Animal grouping and inoculation

For the in vivo experiments, female C57BL/6 mice were randomized into two groups of 5 mice each. To determine the effect of *Plasmodium* infection on MDSCs and Tregs, we infected tumor-bearing mice (seeded with a subcutaneous (s.c.) injection of 5 × 10^5^ LLC cells), with either *Plasmodium yoelli* 17XNL parasitized erythrocytes (LP) or an equivalent number of uninfected erythrocytes (LR). Tumors were harvested 18 days post-inoculation for further analysis (Additional file 1).

### Cell preparation from tumor tissues

Tumor-infiltrating leukocytes were isolated as previously described [29] with modifications. Briefly, tumors were cut into small fragments and digested for 1-3 h at 37 °C with 10X Triple enzyme mix. The cells were washed twice and then depleted of red blood cells. The recovered cells were counted, stained and analyzed for myeloid markers using flow cytometry. All antibodies for flow the cytometry analysis were purchased from eBioscience (San Diego, CA, USA). Antibodies were diluted in PBS/1% BSA, as recommended by the manufacturer, and incubated with the cells for 30 min at 4 °C.

### Sorting of MDSCs and CD8^+^Tcells from tumor tissues

Single-cell suspensions were prepared from tumor tissues and stained as previously described. Ly6G^+^Ly6C^low^ polymorphonuclear cells, Ly6G^−^Ly6C^high^ monocytic cells and CD8^+^T cells were sorted from tumor tissues isolated from tumor-bearing mice injected with uninfected red blood cells (LR), or parasitized erythrocytes (LP) by fluorescence activated cell sorting (FACS, Beckman MoFlo Astrios).

### Flow cytometry

Cells isolated from tumor tissues were blocked with purified CD16/32 antibody for 10 min and then stained for MDSCs using anti-CD11b APC, anti-CD45 PE C-Y7, anti-Ly6G PE, and anti-Ly6C FITC antibodies (eBioscience, San Diego, CA) for 30 min on ice. For Tregs staining, the cells were first stained for extracellular Treg markers (CD4 and CD25) with anti-mouse CD4-FITC,- and anti-mouse CD25-PE for 30 min. The cells were then fixed using the FOXP3/Transcription factor staining buffer set (eBioscience cat.# 00–5523) according to the manufacturer’s protocol. The cells were subsequently washed and stained with DAPI for 10 min before analysis by flow cytometry and Flowjo software V10.

### RNA extraction and QRT-PCR

QRT-PCR was performed using tumor tissues and sorted MDSCs and CD8^+^ Tcells. Flash-frozen tumor tissues were crushed using a sterile pestle and mortar. Total RNA was then isolated using TRIzol (Invitrogen). Isolated total RNA was reverse transcribed into _C_DNA using the Prime Script™ RT-PCR Kit (Takara). The mRNA expression of *VEGF-α*, GM-CSF, M-CSF, G-CSF, SCF, IL-13, IL-14, CCL-17, CCL-22 and IL-6 in the tumor tissues was analyzed by QRT-PCR. For MDSCs and CD8^+^Tcells, the sorted cells were washed with PBS before the isolation of total RNA as described above. The _m_RNA expression of Arginase 1, NOS2, Survivin, IL-10, and S100A9 in MDSCs was analyzed as described above. The expression levels of granzyme B, perforin, IL-2, IFN-g, PD-1, and TNF-α in CD8^+^ Tcells were also analyzed. The relative amount of mRNA in all the analyzed genes was normalized to β-actin. The primer sequences for the cytokines and other proteins analyzed can be found in Additional file 2**:** Table S1.

### Protein extraction and western blotting

Total protein was extracted from the isolated MDSCs, exosomes, exosomes-free plasma and CD8^+^Tcells using lysis buffer (RIPA) and the protein concentration was determined using the BCA method. A total of 15-20 μg of protein was separated by 8–10% SDS-PAGE and then electrophoretically transferred to a PVDF membrane (Millipore).After the membrane was blocked with 5% nonfat milk in TBST solution, it was incubated at 4 °C overnight with primary antibody in blocking solution. The membrane was washed three times with TBST and then incubated at room temperature for 1 h with horseradish peroxidase–conjugated secondary antibody diluted in TBST. Protein bands were visualized by enhanced chemiluminescence (Pierce) and detected using BioImaging Systems (BIO-RAD ChemiDoc™ MP Imaging System, USA). Actin protein was used as a loading control. A list of antibodies used for western blotting can be found in Additional file 3**:** Table S2.

### MDSC ROS production assay

MDSC ROS production was measured using the DCFDA Cellular ROS Detection Assay kit (Abcam, cat # ab113851) according to the manufacturer’s protocol.

### MDSC arginase activity

Arginase activity was measured in cell lysates using the Arginase Activity Assay kit (Abcam, cat.# ab180877) according to the manufacturer’s protocol.

### Isolation of exosomes and exosome-free plasma

Female C57BL/6 mice were randomized into three groups (*n* = 20/group). Mice were infected with only *Plasmodium yoelli* (Py), a combination of LLC and Py (LP), and no infection at all (Naïve). Whole blood was isolated from the respective mice at peak parasitemia (40–45%), into labelled sterile heparinized tubes and subsequently centrifuged at 3000 g for 15 min to remove cells and cell debris. Exosomes were isolated using the Exosomes Isolation kit (SBI System Biosciences cat. #. EXOQ5TM-1) according to the manufacturer’s instructions. Briefly, samples were incubated at room temperature for 30 min, centrifuged at 1500 g for 30 min, and the exosomes-rich fraction was then washed twice with PBS and either stored at − 80 °C for future use or directly used in co-culture experiments. The supernatant remaining after exosome isolation from the Py-infected mice was either stored at -80 °C for future use or directly used in co-culture experiments.

### Characterization of exosomes

Isolated exosomes were diluted in PBS (1000 times) and subsequently used for size measurement and analysis on a NanoSight ns300 Malvern (Worcester, UK). Videos of 60 s for each sample were obtained using the maximum camera gain. Particle size and density were analyzed using the NanoSight particle tracking software (Additional file 4).

### In vitro exosomes and red blood cells treatment

LLC (3 × 105) cells were plated in triplicate overnight in 6 well plates and incubated under standard conditions to allow for cell attachment. Culture medium was removed and cells were washed twice with PBS. Fresh medium was then added to the cells. 0.2 μg/μl of exosomes from different groups and the exosome-free plasma was added to the respective wells and mixed thoroughly. A total of 3 × 10^5^ of either Py-infected RBCs or uninfected RBCs were co-cultured with LLC cells in different wells to determine whether cell-to-cell contact was required for cytokine inhibition. The cells were then incubated for either 24 or 48 h. RNA extraction and QPCR was subsequently performed as earlier described above.

### Statistical analysis

Differences between groups were analyzed for statistical significance using Student’s t-test. *P*-valuesless than 0.05, 0.01, 0.001, and 0.0001 were considered statistically significant, and indicated by *, **, *** and **** respectively, in each figure. Representative results from three independent experiments with similar results are shown. Statistical analysis was performed using GraphPad Prism (version 6).

## Results

### *Plasmodium* infection significantly reduced the proportion of MDSCs in the tumor microenvironment

**Fig. 1:**
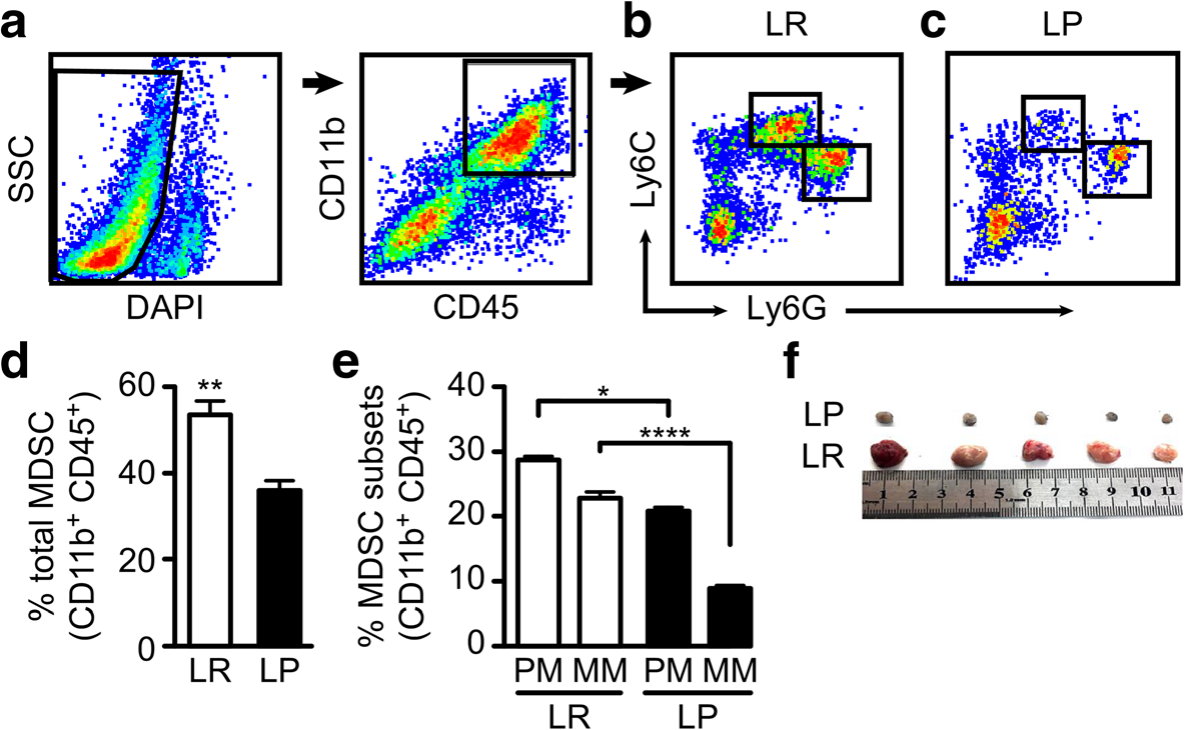
Downregulation of the population of MDSCs in the tumor microenvironment by *Plasmodium*. **a** Gating protocol for the identification of MDSC subsets. **b** Gated polymorphonuclear and monocytic MDSCs in the tumor tissues of uninfected tumor-bearing mice (LR, control). **c** Gated polymorphonuclear and monocytic MDSCs in the tumor tissues of Py-infected tumor-bearing mice (treated, LP). **d** Comparison of the total MDSC population in the tumor tissues of both the treated and the control groups. **e** The relative proportions of polymorphonuclear and monocytic MDSC subsets in the tumor tissues of tumor-bearing mice treated with infected and uninfected RBCs. **f** Tumors harvested from tumor-bearing mice treated or untreated with *Plasmodium* 18 days after tumor inoculation. A total of 50,000 live events was recorded and analyzed per sample. The average number of MDSCs gated out were 2000 cells and 5000 cells for (LP) and (LR) respectively

To examine the MDSC subsets in the tumor microenvironment, single cell suspensions were prepared from the tumor tissues of *Plasmodium. yoelli* 17XNL (Py)-treated and untreated tumor-bearing mice, stained for MDSC markers and analyzed by flow cytometry. After Live/Dead,- and CD45^+^CD11b^+^ gating (Fig.1a), Ly6G^+^Ly6C^low^ polymorphonuclear and Ly6G^−^Ly6C^high^ monocytic cells were gated (Fig.1b&c) and analyzed(*n* = 5/group). Our data indicated a significant reduction in the percentage of MDSCs (Fig.1d) (*P* < 0.01) in the tumor tissues of tumor-bearing mice treated with Py-infected RBCs compared to the control.

Several reports have shown that MDSCs are not evenly distributed in the tumor microenvironment in terms of number or immunosuppressive capability. We wanted to determine the relative proportions of the two major MDSC subsets (P-MDSCs and M-MDSCs) in the tumor microenvironment irrespective of *Plasmodium* treatment. Our flow cytometry analysis indicated a higher proportion of P-MDSCs than M-MDSCs in the tumor tissues of both the untreated and treated mice (Fig.1e). More importantly, there was a more pronounced reduction in the M-MDSCs than the P-MDSCs upon treatment. Because M-MDSCs are believed to play a more prominent immunosuppressive role in the tumor microenvironment than P-MDSCs, it is expected that *Plasmodium* infection might substantially inhibit MDSC-mediated immunosuppression in the tumor microenvironment. Careful observation of the isolated tumor tissues 18 days after tumor inoculation showed a remarkable reduction in the tumor size of Py-treated mice compared to that of the control mice (Fig. 1f).

### *Plasmodium* infection significantly reduced the proportion of Tregs in the tumor microenvironment

**Fig. 2:**
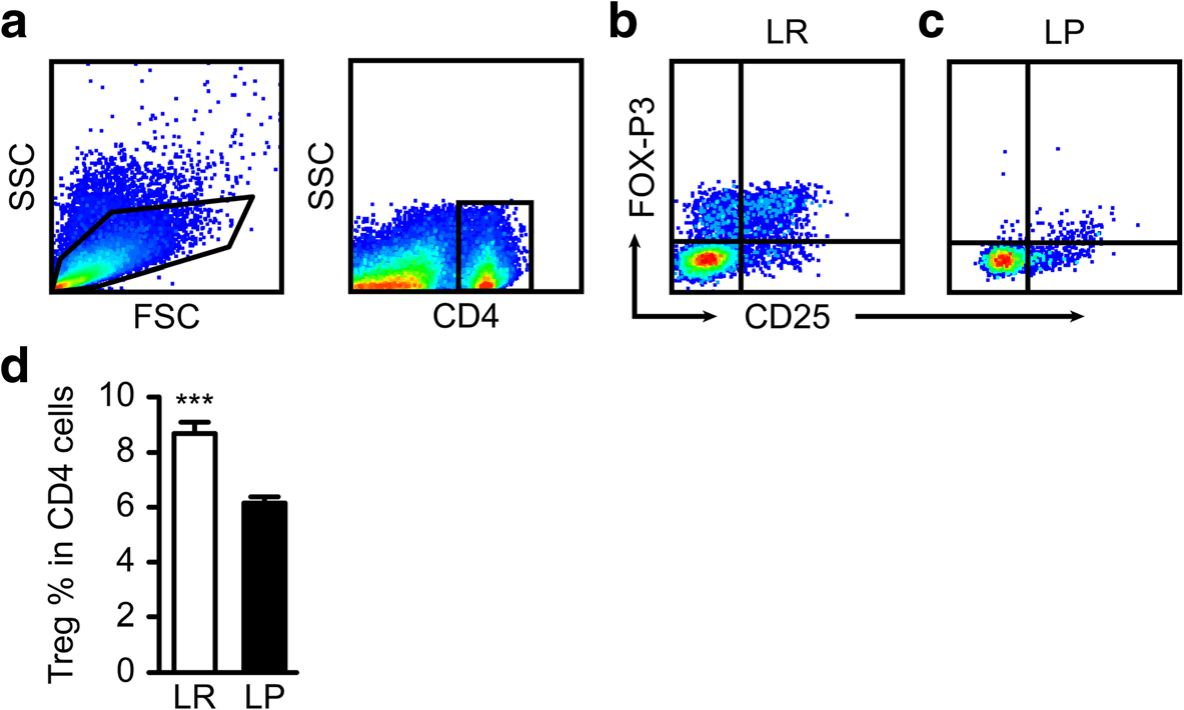
Effects of *Plasmodium* on the population of Tregs in the tumor microenvironment. **a** Gating protocol for CD4^+^ cells. **b** Gated CD25^+^FOXP3^+^ Treg population in untreated tumor tissues. **c** Gated CD25^+^FOXP3^+^ Treg population in treated tumor tissues. **d** The populations of Tregs in the tumors were compared. *Plasmodium* infection significantly downregulated the populations of Tregs in the tumor tissues of tumor-bearing mice compared to the control mice. ***P < 0.001

Our previous work [22] revealed that *Plasmodium* infection significantly upregulated the population of CD4+, CD8+, and granzyme-producing CD8+ Teff cells in the peripheral blood, tumor-draining lymph nodes and tumor tissues in mice. However, it is yet to be understood if *Plasmodium* infection could inhibit immunosuppressive Tregs in the tumor microenvironment. The accumulation of Tregs have been shown to correlate with diminished survival in cancer patients. We therefore investigated whether *Plasmodium* infection could inhibit the accumulation of Tregs in the tumor microenvironment. Single-cell suspensions were prepared from tumor tissues as previously described for MDSCs and subsequently stained for Treg markers. After gating out the DAPI^+^ CD4^+^ subset (Fig. 2a), the population of CD25^+^FOXP3^+^cells (Fig. 2b & c) was gated. Our results indicated a significant reduction in the population of CD25 + FOXP3 + cells in the tumor tissues of Py-treated mice compared to those of the control mice (Fig. 2d) (*P* < 0.001).

### Infection with *Plasmodium* inhibits the expression of key MDSC- and Treg-recruiting cytokines, chemokines and growth factors in the tumor microenvironment

**Fig. 3:**
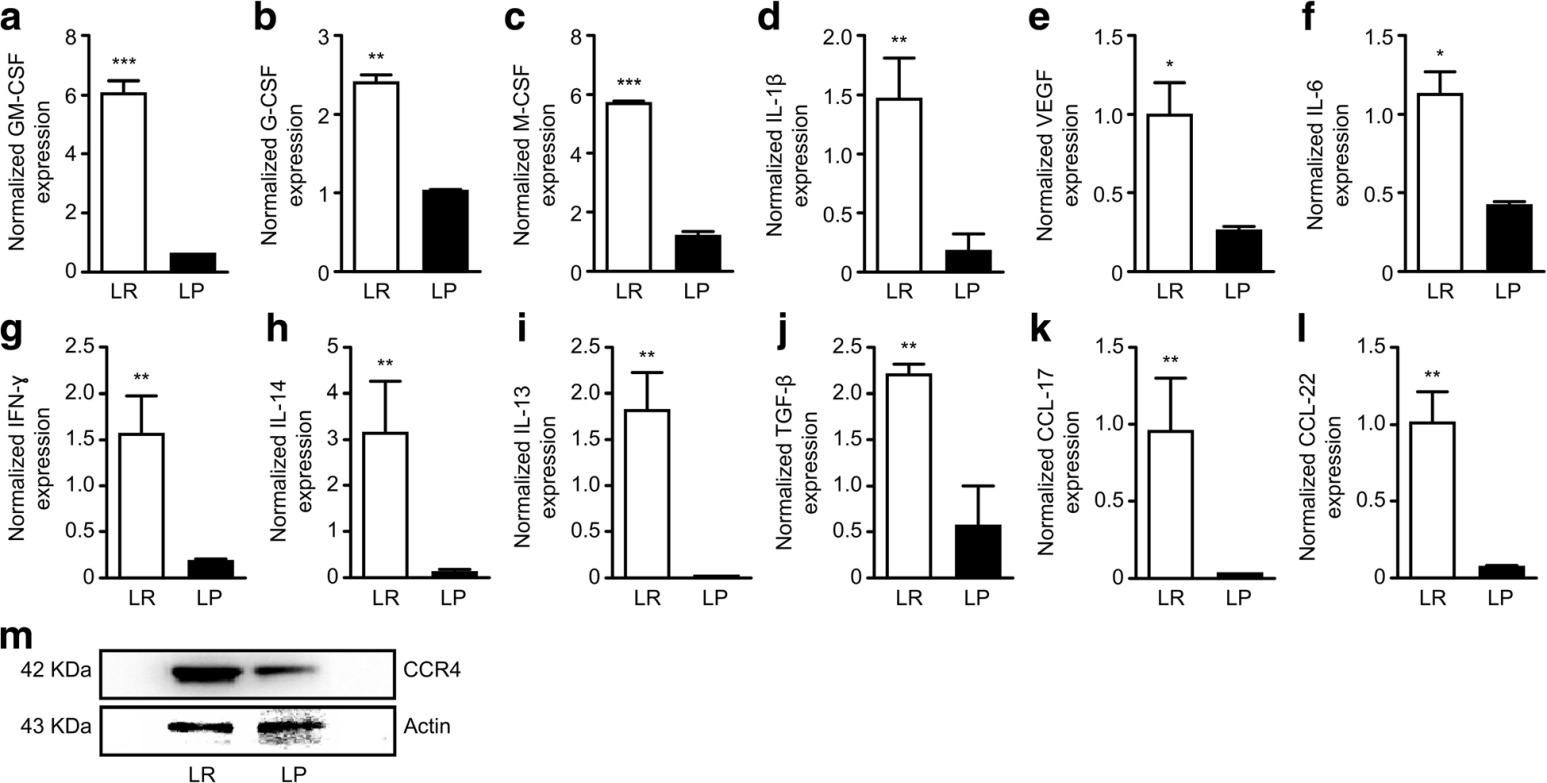
Expression levels of key MDSC- and Treg-recruiting cytokines and chemokines in the tumor tissues assessed by qRT-PCR and western blotting. The relative mRNA expression levels of GM-CSF (**a**), G-CSF (**b**), M-CSF (**c**), IL-1β (**d**), VEGF (**e**), IL-6 (**f**), IFN-g (**g**) IL-14 (**h**), IL-13 (**i**), CCL-17 (**i**) TGF-β (**j**), CCL-17 (**k**), and CCL-22 (**l**) in the tumor tissues of Py-infected tumor-bearing mice compared with those of the control mice. Western blot results of CCR4 protein expression in the tumor tissues (**m**). β-Actin was used as a loading control. *Plasmodium* infection significantly decreased the relative mRNA levels of key MDSC and Treg cytokines and chemokines in the tumor tissues of Py-infected tumor-bearing mice compared to the control mice

MDSCs are recruited to the tumor microenvironment via tumor-secreted cytokines, chemokines and growth factors. To test the effects of *Plasmodium* infection on the secretion of tumor-derived cytokines and chemokines in the tumor microenvironment, we carried out QRT-PCR on tumor samples isolated from tumor-bearing mice treated with either parasitized or unparasitized erythrocytes. Our results showed a significant reduction in the relative mRNA expression of GMCSF (Fig. 3a) (*P* < 0.01), GCSF(Fig. 3b) (*P* < 0.01), MCSF(Fig. 3c) (*P* < 0.001), IL-1β(Fig. 3d) (*P* < 0.01), VEGF(Fig. 3e) (*P* < 0.05), IL-6(Fig. 3f) (*P* < 0.05), IFN-g (Fig. 3g) (*P* < 0.01), IL-14(Fig.3h) (*P* < 0.01), IL-13(Fig. 3i) (*P* < 0.01), TGF-β(Fig. 3j) (*P* < 0.01), CCL-17(Fig. 3k) (*P* < 0.01) and CCL-22(Fig. 3l) (*P* < 0.01) in the Py-treated group compared to the control group suggesting that *Plasmodium* infection could inhibit the generation of immunosuppressive cells in the tumor microenvironment. Furthermore, western blot analysis revealed a reduction in the expression of the CCR4 (Fig.3m) receptor in the tumors of Py-treated mice compared to those of the untreated mice. Several reports have implicated the CCL17/22-CCR4 pathway and tumor-derived TGF-β in Treg recruitment and activation respectively in the tumor microenvironment [30, 31], suggesting that inhibition of the CCL17/22-CCR4 pathway could substantially modulate the tumor accumulation of Tregs.

### MDSCs isolated from the tumors of *Plasmodium*-infected mice showed inhibited phosphorylation of STAT proteins

**Fig. 4:**
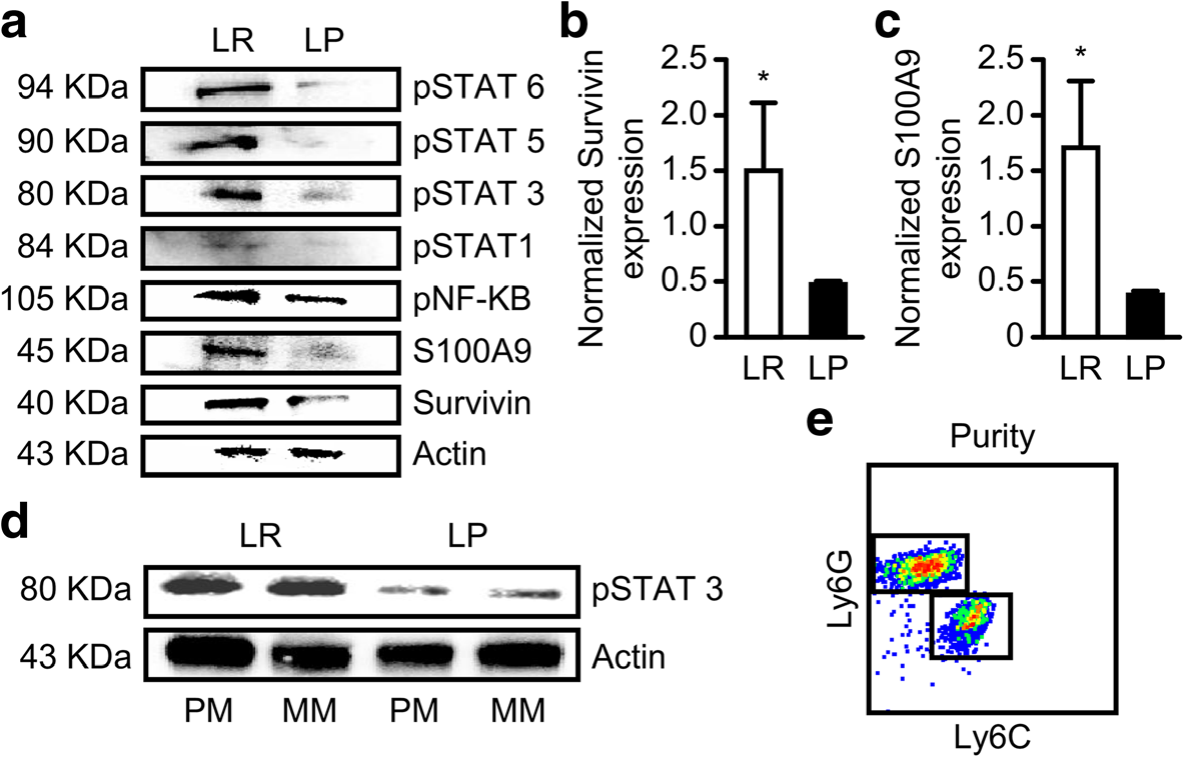
Effects of *Plasmodium* infection on MDSC signal transduction and downstream protein expression. The expression levels of pSTAT1, pSTAT3, pSTAT5, pSTAT6, NF-κB, Survivin, and S100A9 were assessed by qRT-PCR and western blot analysis. **a** The protein expression levels of pSTAT1, pSTAT3, pSTAT5, pSTAT6, NF-KB, Survivin and S100A9 in MDSCs from the infected and control mice, as determined by western blotting. **b**, **c** qRT-PCR analysis of Survivin and S100A9 expression of the sorted MDSCs. **d** The protein expression of pSTAT3 in the two MDSC subsets was assessed. (e) Purity of the sorted MDSCs. β-Actin was used as a loading control for both the qRT-PCR and western blot analysis. *P < 0.05. MM, monocytic MDSC; PM, polymorphonuclear MDSC

Available evidence indicates that the STAT3 pathway is crucial for MDSC differentiation, survival and immunosuppressive functions [32]. Several reports have indicated that activated STAT3 modulates the expression of several target genes involved in cell cycle regulation, angiogenesis, tumor invasiveness and apoptosis [33–35], and that the disruption of STAT3 in myeloid cells alters immunosuppressive cell abundance [36]. We therefore examined whether *Plasmodium* infection could inhibit the phosphorylation of STAT3 and other STAT pathways including those of their downstream proteins – especially those involved in cell survival and immunosuppression. We used western blotting to assess the protein expression levels of p-STAT1, pSTAT3, pSTAT5, pSTAT6, NF-kB, Survivin and S100A9 and QRT-PCR to assess Survivin and S100A9. Our western blot results showed that, compared to the control, *Plasmodium* treatment inhibited the protein expression of p-STAT1, pSTAT3, pSTAT5, pSTAT6, NF-kB, Survivin and S100A9 (Fig.4a). Because the subsets of MDSCs have different immunosuppressive potentials in the tumor microenvironment and because STAT3 is the most crucial factor for MDSC activation, we sorted the individual subsets (P-MDSCs and M-MDSCs) for additional western blot analysis. Our results indicated that *Plasmodium* treatment inhibited the expression of pSTAT3 protein in each of the MDSC subsets analyzed (Fig.4d). Furthermore, we carried out QRT-PCR on the sorted MDSCs to examine the mRNA expression of two downstream proteins (Survivin and S100A9). Our results indicated that *Plasmodium* treatment downregulated the mRNA expression level of Survivin (Fig.4b) (*P* < 0.05), and S100A9 (Fig.4c) (*P* < 0.05) compared to the control. These results suggest that *Plasmodium* infection could modulate MDSC proliferation and survival through inhibition of the STAT pathways.

### MDSCs from the tumors of *Plasmodium-*infected mice have significantly reduced expression of immunosuppressive molecules

**Fig. 5:**
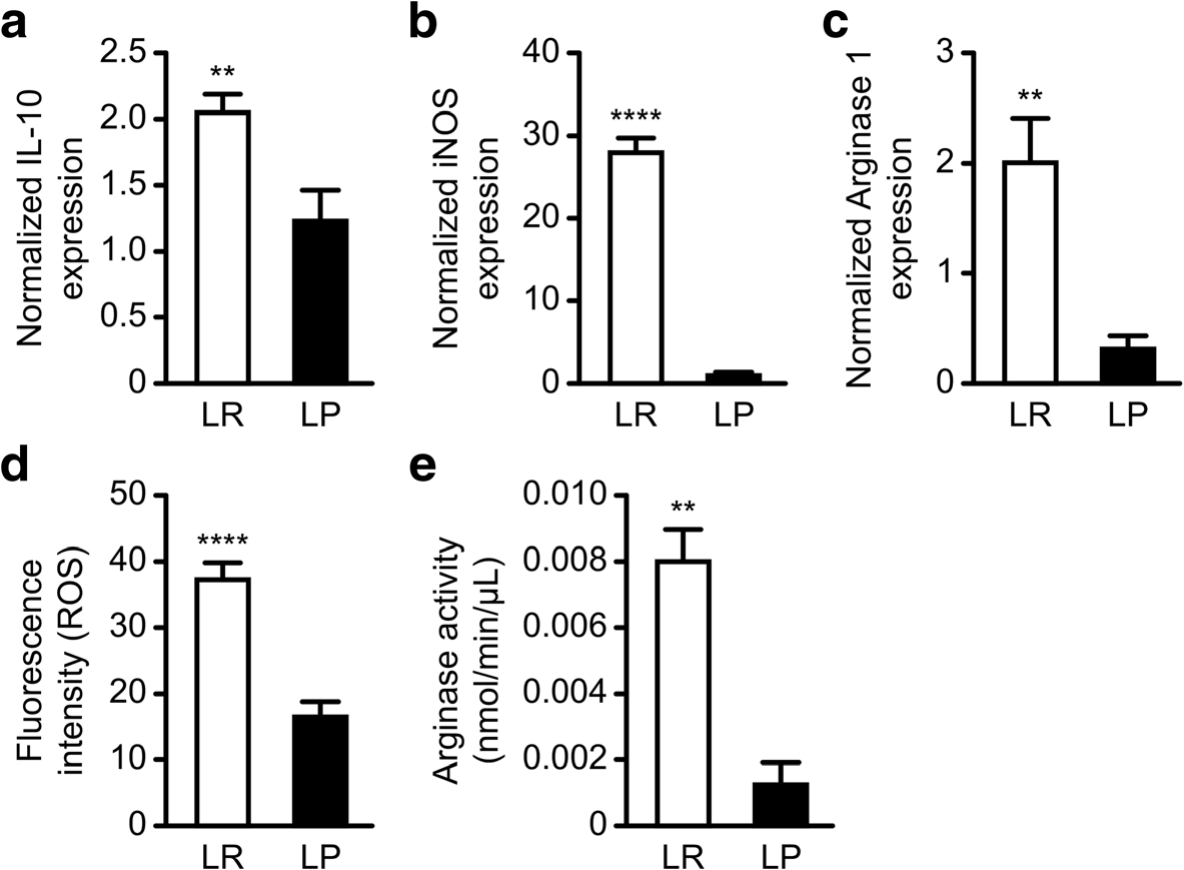
Effects of *Plasmodium* infection on the expression of immunosuppressive molecules by sorted MDSCs. The MDSC expression of IL-10, arginase 1, NOS2, and ROS was assessed by qRT-PCR and functional assays. Relative mRNA expression levels of IL-10 (**a**), NOS2 (**b**), and arginase 1 (**c**) of MDSCs isolated from the tumor tissues of Py-treated and untreated tumor-bearing mice. The levels of ROS (**d**) and arginase activity (**e**) were detected using a DCFDA Cellular ROS Detection Assay Kit (Abcam; cat. # ab113851) and Arginase Activity Assay Kit (Abcam; cat. # ab180877), respectively. **P < 0.01, ****P < 0.0001

**Fig. 6:**
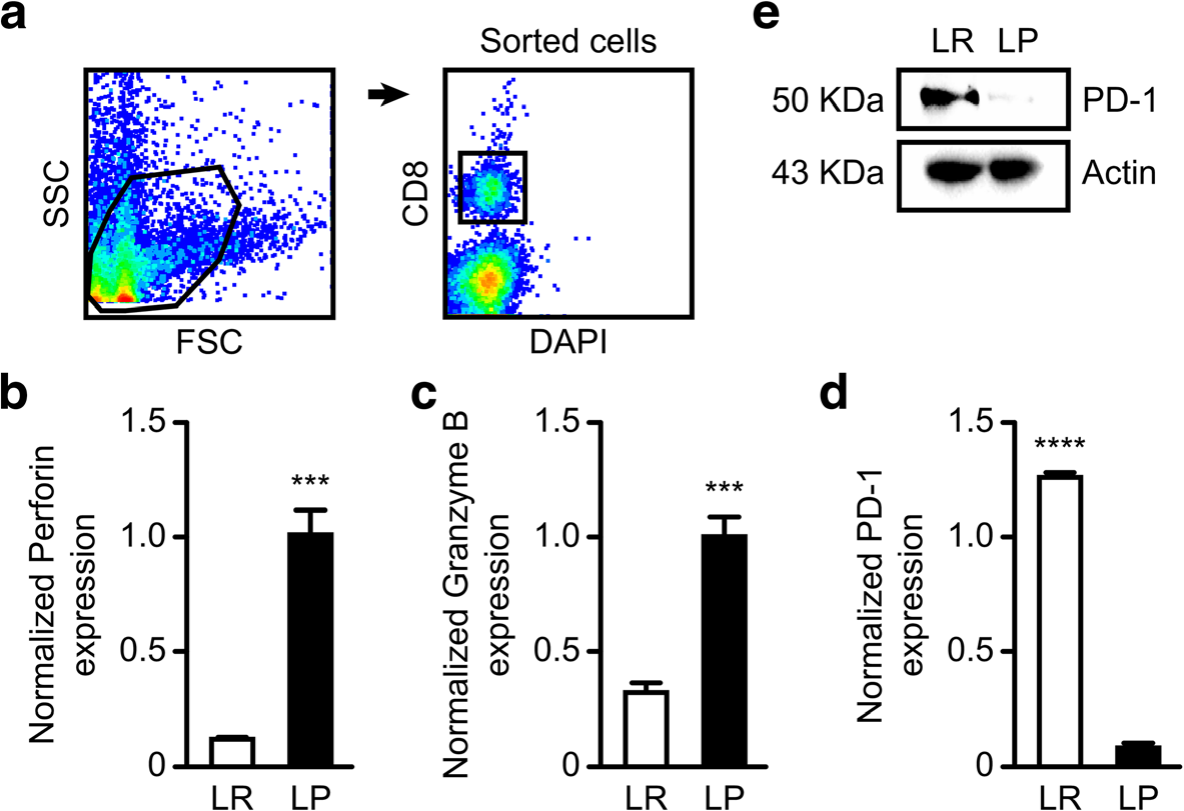
Effects of *Plasmodium* infection on CD8^+^ T cells in the tumor microenvironment. **a** Flow cytometry gating protocol for CD8^+^ T cell sorting. **b** Comparison of the mRNA expression of PD-1 on sorted CD8^+^ T cells. **c** mRNA expression of granzyme B (**d**) and perforin by the sorted CD8^+^ T cells. **e** Protein expression of PD-1 on sorted CD8^+^ T cells determined by western blotting. **P < 0.01

MDSCs are known to inhibit Tcells through the secretion of arginase 1, iNOS, and ROS in the tumor microenvironment [4, 37]. Thus, we next investigated whether *Plasmodium* infection affected the ability of MDSCs to express or secrete T-cell-inhibiting molecules in the tumor microenvironment. Ly6G^+^Ly6C^low^ granulocytic and Ly6G^−^Ly6C^high^ monocytic cells were isolated from the tumor tissues of tumor-bearing mice treated with either parasitized or unparasitized erythrocytes and used for the analysis of immunosuppressive molecules by QRT-PCR and functional assays. Our QRT-PCR results indicated that the mRNA expression levels of IL-10(Fig.5a) (*P* < 0.01), iNOS (Fig.5b) (*P* < 0.0001) and, Arginase 1(Fig.5c) (*p* < 0.01) were downregulated in the treatment group compared to the control group. We further tested the functional status of ROS and Arginase 1 in the isolated MDSCs. We found a significant reduction in ROS activity (Fig.5d) (*P* < 0.01) and Arginase activity (Fig.6e) (*P* < 0.01) in the treated group compared to the control group. These results indicate that *Plasmodium* infection could modulate the immunosuppressive capabilities of MDSCs in the tumor microenvironment by inhibiting the secretion of immunosuppressive molecules.

### CD8^+^ T cells in the tumor tissues of *Plasmodium*-treated mice expressed high levels of cytotoxic molecules and low levels of PD-1

CD8^+^ Tcells effect tumor killing in two ways; (i) through the secretion of cytotoxic molecules (granzyme B and perforin) and, (ii) through the secretion of proinflammatory cytokines (IL-2, TNF-α, and IFN-g). We therefore examined whether the inhibition of MDSCs and Tregs in the tumor microenvironment could promote the tumor cell-killing ability of CD8^+^ Tcells. We isolated CD8^+^T cells from the tumors and carried out QRT-PCR. Interestingly, we found that the CD8^+^ Tcells from the tumors of *Plasmodium*-treated mice expressed remarkably higher levels of perforin and granzyme B (Fig. 6b &c). However, we observed that *Plasmodium* infection did not promote the ability of CD8^+^T cells to produce proinflammatory cytokines (IL-2, IFN-g, and TNF-α; data not shown). Because the B7-H1(PD-L1)/PD-1 signaling pathway is believed to play a key role in T cell exhaustion [38], we analyzed the PD-1 expression of sorted CD8^+^ Tcells by QRT-PCR and western blotting. We found a remarkable reduction in the expression of PD-1 on CD8^+^ Tcells in the tumor tissues at both the mRNA (Fig. 6d) and protein (Fig. 6e) levels. These results suggest that *Plasmodium* infection could promote CD8^+^T cell-mediated tumor cell-death in the tumor microenvironment through the upregulation of granzyme B and perforin while preventing inhibition through the PD-1/PD-L1 signaling pathway.

### *Plasmodium* infection inhibits tumor cytokines and chemokines secretions through exosomes-like vesicles

**Fig. 7:**
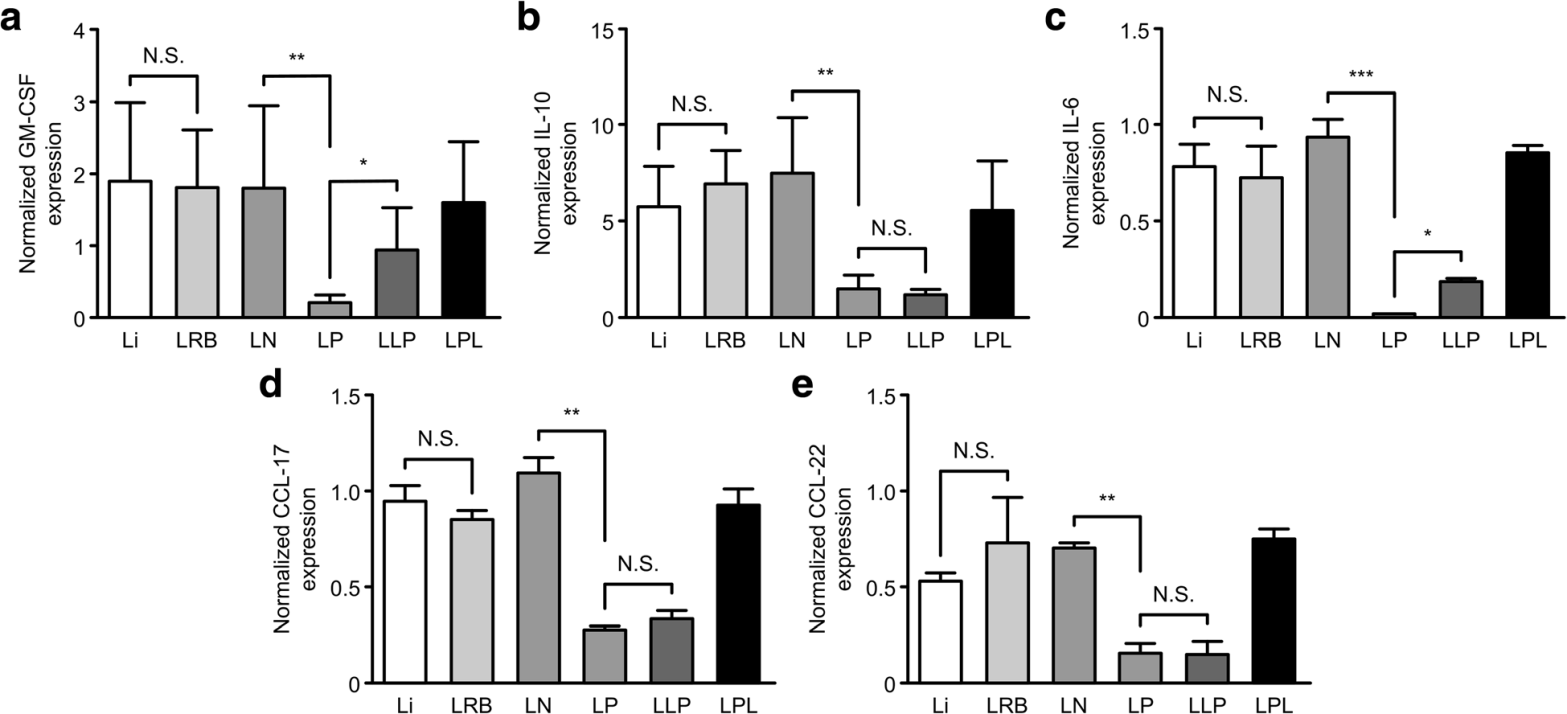
*Plasmodium* infection inhibits tumor cytokines and chemokines secretions through exosomes-like vesicles. To determine whether or not cell-to-cell contact was required for the inhibition of tumor cytokines, we co-cultured LLC cells with either infected RBCs (Li), uninfected RBCs (LRB), exosomes isolated from uninfected mice (LN), exosomes isolated from Py-infected mice (LP), exosomes isolated from tumor-bearing mice infected with Py (LLP), or plasma from Py-infected mice after exosomes have been isolated (LPL). Cells were cultured for either 24 or 48 h and subsequently harvested for RNA extraction and QPCR analysis. Our QPCR results suggest that infected RBCs does not communicate with tumor cells through cell-to-cell contact. Exosome-like vesicles significantly inhibited tumor release of GMCSF(**a**), IL-10(**b**), IL-6(**c**), CCL-17(**d**), and CCL-22(**e**) at the mRNA levels

*Plasmodium sporozoites* injected into the blood stream have demonstrated high affinity for liver cells due to the availability of highly sulfated heparan sulphate proteoglycans (HSPG) [39]. This therefore suggests that interactions between *Plasmodium* and the tumor could be mediated via the infected red blood cells or through the release of microvesicles. We asked if the inhibition of tumor cytokine and chemokine secretion is mediated by cell-to-cell contact or through the release of microvesicles. Our results indicated that *Plasmodium* infection significantly inhibits the tumor secretions of GMCSF, IL-10, IL-6, CCL-17, and CCL-22 in vitro (Fig. 7a-f) through exosomes-like vesicles released by infected red blood cells.

## Discussion

Tumor growth is often associated with both the stimulation of antitumor T cell responses and a parallel induction of immune suppression. The balance of these two events determines, to a large extent, the prognosis of the disease. In cancer patients, the tumor-specific Tcell response has been found to correlate with improved outcomes [40–44]. However, the tumor-specific immune response is compromised by the recruitment of immunosuppressive cellular and molecular mechanisms within the tumor microenvironment. Several reports have indicated that immunosuppressive cells mobilized into the tumor microenvironment by tumor-derived factors and cytokines are a major cause of the failure of novel immunotherapies and chemotherapies [45–47]. Among these cellular infiltrates, MDSCs and Tregs are believed to play a major role in the suppression of antitumor immune responses [48–50]. Srivastava *et.al*, reported that targeting MDSCs could augment the antitumor immune response against lung cancer [51]. We previously reported that *Plasmodium* infection could induce potent innate and adaptive antitumor immune responses in a lung cancer mouse model [22], but the effect of *Plasmodium* infection on the immunosuppressive abilities of MDSCs and Tregs in the tumor microenvironment is not yet known.

In the current study, we provide evidence that *Plasmodium* infection could inhibit the recruitment and activation of MDSCs and Tregs in the tumor microenvironment in a variety of ways, including the inhibition of STAT phosphorylation (Additional file 5). We demonstrated that *Plasmodium* infection downregulated the secretion of several MDSC- and Treg- recruiting cytokines and growth factors in the tumor microenvironment which is consistent with the reduction in the proportion of MDSCs and Tregs in the tumor tissues of the Py-treated tumor-bearing mice (Figs. 1 & 2). The significant reduction in the proportion of MDSCs and Tregs in the tumor tissues of the treatment group may partially explain the remarkable reduction in tumor size observed (Fig. 1f), potentially due to enhanced T-cell–mediated tumor cell-death orchestrated by the inhibition of MDSC- and Tregs-mediated immune suppression. This finding corroborates other findings demonstrating the efficacy of strategies targeting MDSCs and Tregs [52, 53].

We further observed that P-MDSCs out-numbered M-MDSCs in the tumor tissues of both the treated and untreated tumor-bearing mice (Fig.1e). Although M-MDSCs are believed to play a dominant role in the tumor microenvironment, the relatively higher proportion of P-MDSCs may be due to the ability of M-MDSCs to rapidly differentiate into tumor-associated macrophages(TAMs, reviewed in [12]).

A major pathway in the recruitment of Tregs into the tumor microenvironment is the CCL17/22-CCR4 pathway. We observed that *Plasmodium* infection significantly downregulated the expression of the chemokines CCL17 and CCL22 as well as their receptor, CCR4 in the tumor microenvironment (Fig. 3k-m) further validating the reduction in the proportion of Tregs in the tumor tissues.

Available evidence points to the fact that MDSCs and Tregs contribute significantly to immune regulation in the tumor microenvironment and impair the long-term efficacy of current combination therapies for lung cancers [54]. The activation of STAT signaling in myeloid cells promotes cell proliferation and prevents apoptosis through the expression of Survivin, S100A9, Bcl-xL, c-myc, or cyclin D1 [55]. In our study, MDSCs isolated from the tumors of Py-treated mice showed significant reduction in the expression of phosphorylated STAT proteins, S100A9, and Survivin at both the mRNA and protein levels, further explaining their reduced proportion in the tumor tissues.

One of the ways MDSCs contribute to immunosuppression in the tumor microenvironment is by generating ROS, Arginase 1, free radicals and cytokines to suppress host CD4+ and CD8+ Tcell responses [8, 56, 57].It is believed that Arginase and iNOS function synergistically in MDSCs to inhibit antigen-specific T cell response [58, 59]. It has been demonstrated that the production of ROS by MDSCs could downregulate the ζ-chain on Tcells and induce Tcell tolerance [46, 60]. The ability of *Plasmodium* infection to perturb ROS generation, inhibit arginase activity, and downregulate iNOS and IL-10 expression clearly demonstrates the potential benefits of the *Plasmodium*-mediated targeting of immunosuppressive cells in the tumor microenvironment.

CD8^+^T cell-mediated immunity plays a vital role in limiting cancer initiation and progression. At steady state, CD8^+^T cells can eliminate cancer cells by releasing cytotoxic granules containing granzyme and perforin and at the same time promote inflammation by releasing proinflammatory cytokines [61]. IFNγ released by CD8^+^T cells promotes the tumor expression of MHC class 1 antigens thereby making tumors susceptible to immune cell detection and elimination [62]. Interestingly, our results indicated that sorted CD8^+^ cytotoxic Tcells expressed remarkably high levels of granzyme B and perforin indicating a high tumor cell-killing potential. However, there were no observable increases in the expression of the proinflammatory cytokines tested (IL-2, TNF-α, and IFNγ) within the tumor tissues. Although it was impossible for us to measure the cytokine levels in the tumor microenvironment at the early days of infection(1–7 days post infection), our current results may be a reflection of our earlier report that *Plasmodium* infection upregulates the production of proinflammatory cytokines(IL-2, IFNγ and TNF-α) in peripheral blood during the early stage of infection, peaks at day 7, and declines to a minimal level 14 days post infection [63]. Because the tumor samples were harvested 18 days after *Plasmodium* injection, this timing may account for the reduced expression of proinflammatory cytokines by CD8^+^T cells in the tumor microenvironment.

It is believed that intratumoral T cells demonstrate exhaustion by expressing high levels of inhibitory surface molecules such as PD-1, CD160, CTLA-4, LAG-3, TIM-3 and BTLA that can prevent T cell activation. Among those inhibitory molecules, researchers believe that those of the PD-1/PD-L1 signaling pathway plays a key role in CD8^+^Tcell functional exhaustion. We therefore tested the expression of PD-1 in the sorted CD8^+^Tcells by QRT-PCR and western blotting. Interestingly, our results indicated a significant reduction in the expression of PD-1 on CD8^+^ T cells (Fig. 6d), highlighting the potential of utilizing *Plasmodium* infection to develop effective immunotherapies.

The asexual phase of the *Plasmodium* life cycle is initiated by the injection of sporozoites into the mammalian host. Sporozoites are known to rapidly invade the liver, develop in the liver and are released as merozoites that invades red blood cells within minutes [64]. Therefore, interactions between *Plasmodium* and tumor cells can only be mediated through the infected red blood cells or through microvesicles secreted from infected red blood cells. Our co-culture experiments revealed that infected and uninfected red blood cells did not inhibit tumor cytokine and chemokines secretion, suggesting that cell-to-cell contact was not involved in this inhibition. In contrast, exosomes from infected red blood cells significantly inhibited the secretion of cytokines and chemokines from the tumor cells at the messenger RNA levels when compared to the controls. The release of exosome-like vesicles from cells under stress conditions have since been reported in several papers. Interestingly, *Plasmodium* falciparum-infected RBCs have been demonstrated to directly communicate through exosome-like vesicles capable of delivering genes [65].

Further work should focus on the specific proteins, molecules, microRNAs and/or long noncoding RNAs in the exosomes responsible for these *Plasmodium*-mediated effects with the aim of developing effective immunotherapeutic strategies against lung cancer.

## Conclusions

In conclusion, we report that *Plasmodium* infection inhibits the accumulation of immunosuppressive MDSCs and Tregs in the tumor microenvironment in a murine lung cancer model. The inhibition of MDSC STAT phosphorylation, the downregulation of immunosuppressive molecules, the elevated expression of granzyme B and perforin by CD8^+^ Tcells and the inhibition of PD-1 expression on CD8^+^Tcells clearly demonstrate the relevance of these findings and their merit for further investigation. We further demonstrated that *Plasmodium* infection inhibits tumor secretion of cytokines and chemokines through the release of exosome-like vesicles rather than through cell-to-cell contact. This study advances our understanding of the effect of *Plasmodium* infection in enhancing the host immune response to lung cancer and provides insight into the development of potent immunotherapeutic strategies. Subsequent studies will focus on the identification of specific molecules in the exosomes that are responsible for the inhibition of the cytokine signaling in tumor cells.

## Additional files

Additional file 1: **Figure S2.** Schematic diagram summarizing the experimental design. (TIF 268 kb)

Additional file 2: **Table S1.** List of primers used for qRT-PCR. (DOCX 161 kb)

Additional file 3: **Table S2.** List of antibodies used for western blotting and the companies from which they were obtained. (DOCX 12 kb)

Additional file 4: **Figure S3.** Characterization of exosomes by Nanosight. (a)Analysis of the sizes of exosomes isolated from the plasma of mice infected with Py (Py ex) and uninfected mice (N ex) (**P < 0.01). (b) Analysis of the particles number of exosomes isolated from the plasma of mice infected with Py (Py ex) and uninfected mice (N ex). (*P < 0.05). (C) Representative graph obtained from Nanosight data. (TIF 337 kb)

Additional file 5: **Figure S1.** Graphical abstract of *Plasmodium* modulation of MDSC- and Treg-mediated regulation of CD8^+^ T cells in the tumor microenvironment. Tumor cytokines and chemokines inhibit the differentiation of myeloid cells in the tumor microenvironment, leading to the accumulation of MDSCs. The binding of cytokines to their receptors on MDSCs triggers the phosphorylation of several signal transduction molecules and the activation of transcription, resulting in the expression of downstream proteins. MDSCs express arginase 1, ROS, and iNOS, which inhibit cytotoxic T lymphocytes. The expression of anti-apoptosis proteins, Survivin and S100A9 enables MDSC proliferation and accumulation. Tumor-secreted cytokines convert naïve CD4^+^ T cells to Tregs in the tumor microenvironment. Tregs further inhibit CTLs by releasing molecules such as IL-10 and TGF-β. (G, cytokines; GR, cytokine receptors). *Plasmodium* infection inhibits tumor-derived cytokine and chemokine secretion in the tumor microenvironment, thereby inhibiting the conversion of myeloid cells to MDSCs, the expression of downstream proteins, the conversion of naïve CD4^+^ T cells to Tregs, and the expression of PD-1 on cytotoxic T cells. (TIF 1593 kb)

## Abbreviations

DC: dendritic cells
iNOS: inducible nitric oxide synthase
LLC: Lewis lung cancer cells
MDSCs: myeloid derived suppressive cells
M-MDSC: monocytic myeloid derived suppressor cells
MØ: macrophages
NK: natural killer cells
PD-1: programmed cell death 1
PD-L1: programmed cell death ligand 1
P-MDSC: polymorphonuclear myeloid derived suppressive cells
Py: *plasmodium* yoelli
qPCR/QRT-PCR: quantitative PCR
ROS: reactive oxygen species
STAT: signal transducer and activator of transcription
TAM: tumor-associated macrophages
Tregs: regulatory T cells
