# Supplementary figures and images for: Plasmodium infection inhibits the expansion and activation of MDSCs and Tregs in the tumor microenvironment in a murine Lewis lung cancer model
Source: Cell Commun Signal. 2019 Apr 12;17:32. doi: 10.1186/s12964-019-0342-6 (PMC6461823; doi:10.1186/s12964-019-0342-6)

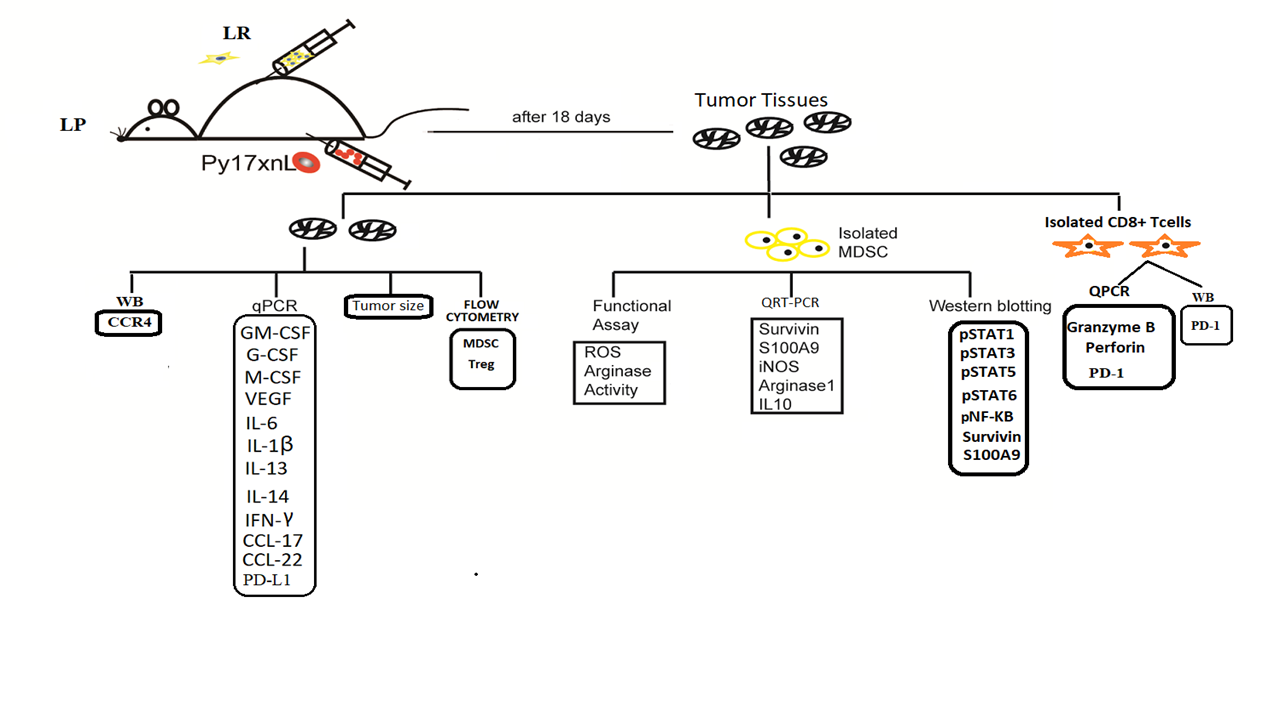

Supplement: Supplementary file 1 — Figure S2. Schematic diagram summarizing the experimental design. (TIF 268 kb) [file 12964_2019_342_MOESM1_ESM.tif]

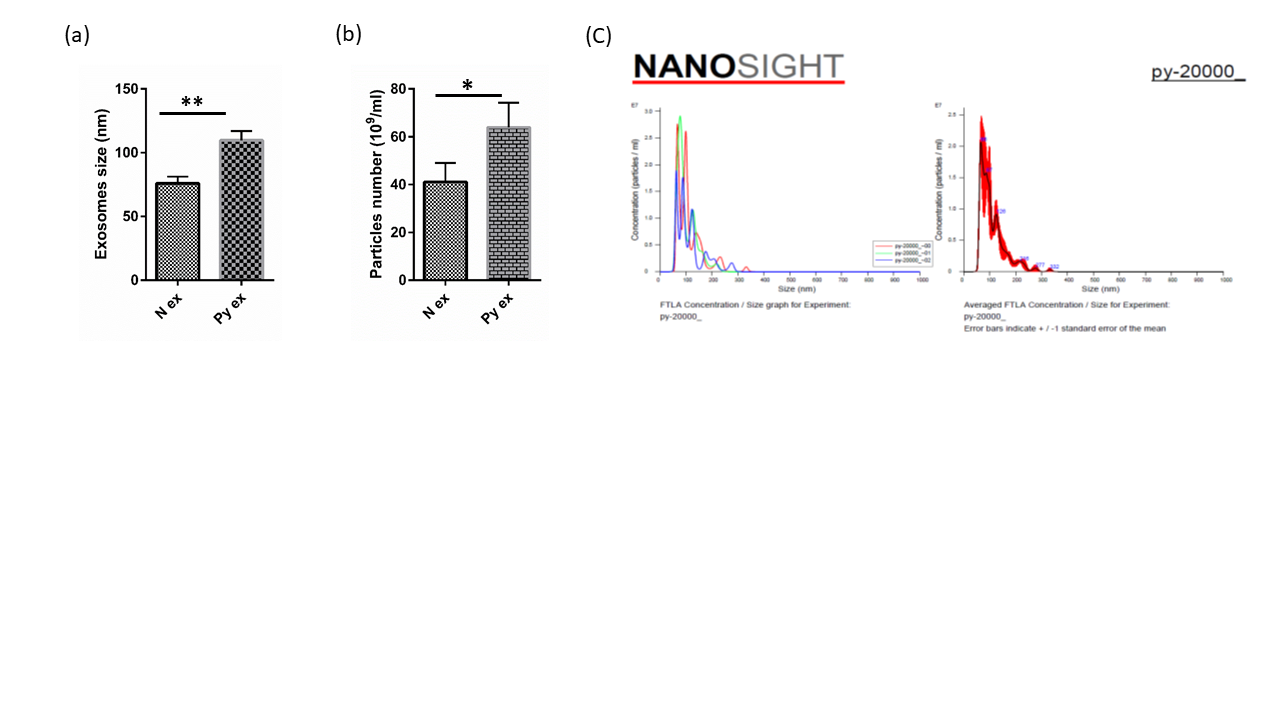

Supplement: Supplementary file 4 — Figure S3. Characterization of exosomes by Nanosight. (a)Analysis of the sizes of exosomes isolated from the plasma of mice infected with Py (Py ex) and uninfected mice (N ex) (**P < 0.01). (b) Analysis of the particles number of exosomes isolated from the plasma of mice infected with Py (Py ex) and uninfected mice (N ex). (*P < 0.05). (C) Representative graph obtained from Nanosight data. (TIF 337 kb) [file 12964_2019_342_MOESM4_ESM.tif]

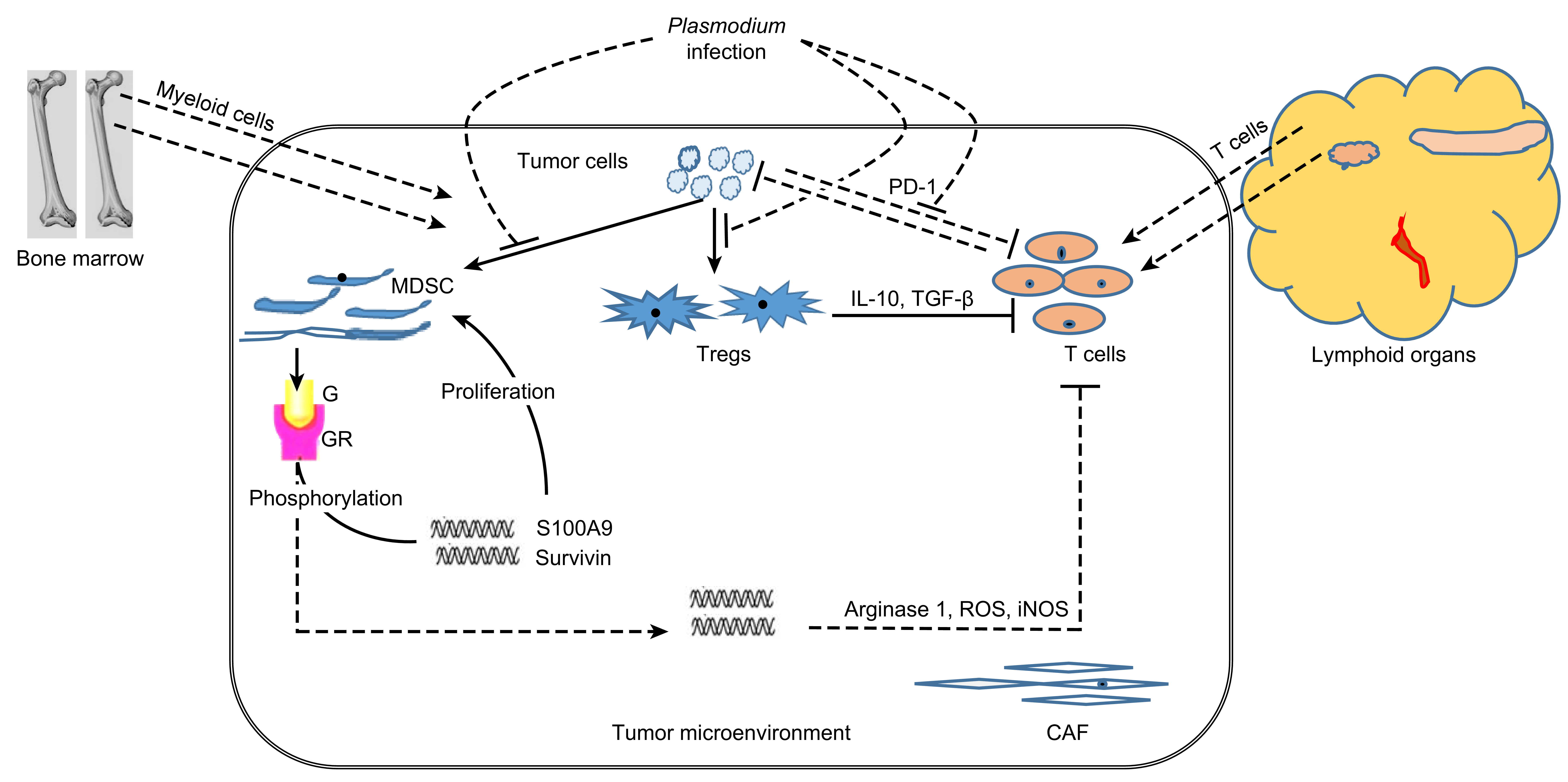

Supplement: Supplementary file 5 — Figure S1. Graphical abstract of Plasmodium modulation of MDSC- and Treg-mediated regulation of CD8+ T cells in the tumor microenvironment. Tumor cytokines and chemokines inhibit the differentiation of myeloid cells in the tumor microenvironment, leading to the accumulation of MDSCs. The binding of cytokines to their receptors on MDSCs triggers the phosphorylation of several signal transduction molecules and the activation of transcription, resulting in the expression of downstream proteins. MDSCs express arginase 1, ROS, and iNOS, which inhibit cytotoxic T lymphocytes. The expression of anti-apoptosis proteins, Survivin and S100A9 enables MDSC proliferation and accumulation. Tumor-secreted cytokines convert naïve CD4+ T cells to Tregs in the tumor microenvironment. Tregs further inhibit CTLs by releasing molecules such as IL-10 and TGF-β. (G, cytokines; GR, cytokine receptors). Plasmodium infection inhibits tumor-derived cytokine and chemokine secretion in the tumor microenvironment, thereby inhibiting the conversion of myeloid cells to MDSCs, the expression of downstream proteins, the conversion of naïve CD4+ T cells to Tregs, and the expression of PD-1 on cytotoxic T cells. (TIF 1593 kb) [file 12964_2019_342_MOESM5_ESM.tif]
