# Supplementary material for: Plasmodium infection inhibits the expansion and activation of MDSCs and Tregs in the tumor microenvironment in a murine Lewis lung cancer model
Source: Cell Commun Signal. 2019 Apr 12;17:32. doi: 10.1186/s12964-019-0342-6 (PMC6461823; doi:10.1186/s12964-019-0342-6)
Supplement: Supplementary file 2 — Table S1. List of primers used for qRT-PCR. (DOCX 161 kb) [file 12964_2019_342_MOESM2_ESM.docx]

| **Name** | **Nucleotides** |
| --- | --- |
| GM-CSF | 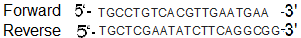 |
| G-CSF | 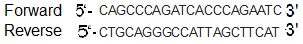 |
| M-CSF | 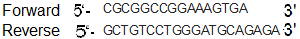 |
| IL-1β | 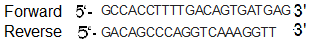 |
| VEGF | 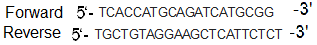 |
| IL-6 | 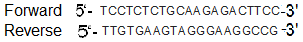 |
| IL-14 | 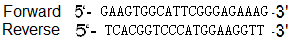 |
| IL-13 | 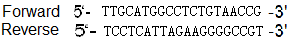 |
| CCL-17 | 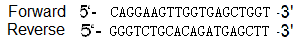 |
| CCL-22 | 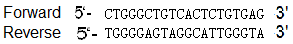 |
| IFN-g | 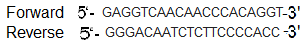 |
| TGF-β | 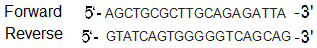 |
| IL-10 | 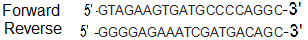 |
| Arginase 1 | 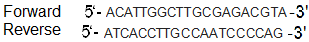 |
| iNOS | 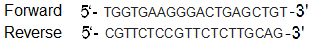 |
| Actin | 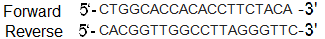 |
| Survivin | 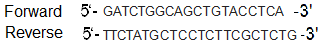 |
| S100A9 | 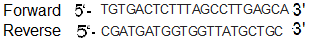 |
| Granzyme B | 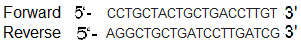 |
| Perforin | 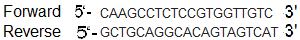 |
