# Supplementary material for: Plasmodium infection inhibits the expansion and activation of MDSCs and Tregs in the tumor microenvironment in a murine Lewis lung cancer model
Source: Cell Commun Signal. 2019 Apr 12;17:32. doi: 10.1186/s12964-019-0342-6 (PMC6461823; doi:10.1186/s12964-019-0342-6)
Supplement: Supplementary file 3 — Table S2. List of antibodies used for western blotting and the companies from which they were obtained. (DOCX 12 kb) [file 12964_2019_342_MOESM3_ESM.docx]

| **S/N** | **Name of Antibody** | **Cat. No.** | **Name of Company** |
| --- | --- | --- | --- |
| 1 | Phospho STAT1 antibody | AF3300 | Affinity |
| 2 | Phospho-Stat3 antibody | #4113 | Cell Signal |
| 3 | Phospho STAT5 antibody | AF3305 | Affinity |
| 4 | Phospho STAT6 antibody | AF3302 | Affinity |
| 5 | Phospho NF-KB antibody | AF3219 | Affinity |
| 6 | Anti-mouse secondary antibody | S0002 | Affinity |
| 7 | Anti-rabbit secondary antibody | S0001 | Affinity |
| 8 | Anti-mouse CD8 antibody APC | 17-0081-82 | Thermofisher |
| 9 | Rabbit anti-mouse polyclonal to S100A9 | ab75478 | Abcam |
| 10 | Rabbit anti-mouse polyclonal to Survivin | ab469 | Abcam |
| 11 | Anti-PD-L1 antibody | ab233482 | Abcam |
| 12 | Anti-CCR4 antibody | NB100-56336 | Novus Biologicals |
